# Alkalinity of diverse water samples can be altered by mercury preservation and borosilicate vial storage

**DOI:** 10.1038/s41598-021-89110-w

**Published:** 2021-05-11

**Authors:** Benjamin Mos, Ceylena Holloway, Brendan P. Kelaher, Isaac R. Santos, Symon A. Dworjanyn

**Affiliations:** 1 National Marine Science Centre, Faculty of Science and Engineering, Southern Cross University, Coffs Harbour, NSW Australia; 2 Department of Marine Sciences, University of Gothenburg, Gothenburg, Sweden

**Keywords:** Carbon cycle, Biogeochemistry, Climate sciences, Environmental sciences, Hydrology, Limnology, Ocean sciences, Chemistry, Analytical chemistry, Chemical safety, Environmental chemistry, Inorganic chemistry, Organic chemistry

## Abstract

We compared the effects of preservation and storage methods on total alkalinity (A_T_) of seawater, estuarine water, freshwater, and groundwater samples stored for 0**–**6 months. Water samples, untreated or treated with HgCl_2_, 0.45 µm filtration, or filtration plus HgCl_2_, were stored in polypropylene or borosilicate glass vials for 0, 1, or 6 months. Mean A_T_ of samples treated with HgCl_2_ was reduced by as much as 49.1 µmol kg^−1^ (1.3%). Borosilicate glass elevated A_T_, possibly due to dissolving silicates. There was little change in A_T_ of control and filtered samples stored in polypropylene, except for untreated groundwater (~ 4.1% reduction at 6 months). HgCl_2_ concentrations of 0.02–0.05% reduced the A_T_ of fresh, estuarine, and ground water samples by as much as 35.5 µmol kg^−1^ after 1 month, but had little effect on the A_T_ of seawater. Adding glucose as a carbon source for microbial growth resulted in no A_T_ changes in 0.45 µm-filtered samples. We suggest water samples intended for A_T_ analyses can be filtered to 0.45 µm, and stored in polypropylene vials at 4 °C for at least 6 months. Borosilicate glassware and HgCl_2_ can be avoided to prevent analytical uncertainties and reduce risks related to use of Hg^2+^.

## Introduction

Total alkalinity (A_T_) is a measure of the capacity of water to buffer against changes in acidity. Interest in alkalinity measurements has increased in recent years as research into the global carbon cycle and anthropogenic climate change has intensified. For instance, alkalinity measurements are required to understand the impacts of ocean acidification on marine organisms^1^, resolve feedbacks among aquatic and atmospheric carbon pools^2^, quantify critical processes such as coral reef calcification^3^, model biological and non-biological responses to global warming and increased CO_2_ levels^4^, and assess novel climate adaptation strategies^5^. Fundamental to the application of A_T_ is its accurate measurement^6^.

Accuracy of A_T_ measurements relies on the methods used to preserve and store samples prior to analysis. These methods are well established for seawater samples^7,8^. There is, however, a paucity of studies comparing the effectiveness of preservation and storage methods for non-oceanic water samples, particularly for samples collected from groundwater or brackish ecosystems. Only three studies have examined aspects of preservation or storage methods for freshwater A_T_ samples^9–11^. It is important that storage and preservation methods are investigated for non-marine water samples given there is growing interest in quantifying the role of estuarine, freshwater, and groundwater systems in the global carbon cycle^12,13^.

For logistical reasons, water samples are typically collected and stored for hours to months prior to A_T_ analysis. It is necessary to inhibit biological activity in samples because biogeochemical processes can alter A_T_^14^. The conventional method to inhibit biological activity in stored water samples is the addition of a saturated HgCl_2_ solution, which was first developed for water samples stored for analyses of N, P, and Si^15,16^. Arguably, the use of HgCl_2_ became established as the primary preservation method for A_T_ samples after 2007 when standard operating procedures (SOP) for analyses of seawater carbonate chemistry were described^7^. There is, however, substantial concern about global mercury levels and pollution^17^ including the use of HgCl_2_ for water preservation^18^, and the applicability of HgCl_2_ to samples other than seawater. The toxicity and environmental persistence of Hg^2+^ presents a health risk for researchers and requires substantial costs for safe handling and disposal^19,20^. In addition, failure to account for the diluting effects of added HgCl_2_ solutions on A_T_ is a potential source of error in analyses^7^. The drawbacks of HgCl_2_ have driven the search for low-cost, safer, and more environmentally benign alternatives^18^. Filtration does not affect the A_T_ of alpine freshwater^9^, pond water^11^, or seawater^21–23^. Filtration is as or more effective than HgCl_2_ in preserving stable isotope compositions (*δ*^13^C) in dissolved inorganic carbon^24,25^. However, there has been no systematic comparison of the efficacy of HgCl_2_ and filtration in preserving A_T_.

In addition to biological activity, A_T_ can be influenced by the material in which water samples are stored. SOP guidelines for seawater analysis recommend samples are collected and stored in borosilicate glass bottles with ground-glass stoppers^7^. Seawater A_T_ concentrations can be elevated by leachates from soda-lime glassware^8^, with the potential to introduce additional errors into calculations of carbonate chemistry parameters via inaccurate A_T_ values^23^. Laboratory borosilicate glassware leach acid-neutralising silicates and phosphates at varying rates depending on the pH, temperature, and salinity of the water^26–28^. The capacity of leachates from borosilicate glass storage vessels to alter A_T_ remains unclear. The only study to test the effects of borosilicate glass vials on A_T_ used standards stored in borosilicate glass bottles as benchmarks, and removed outliers that might have been attributable to leachates^8^. Groundwater may be particularly susceptible to overestimation of A_T_ due to variable pH and complex chemical composition altering the rate of borosilicate glass dissolution (e.g.^29,30^), but this has not been tested. With growing interest in quantifying the contribution of submarine groundwater discharge to the marine carbon cycle^31^, it is important to refine the techniques for measuring A_T_ in groundwater. Polyethylene and polypropylene have shown promise as inert, inexpensive, and robust alternatives to glass for storing drinking water and seawater samples prior to A_T_ analysis^8,10^.

In this study, we tested the efficacy of practical, low-cost, and safer alternatives to the use of HgCl_2_ and borosilicate glass for A_T_ preservation and storage of seawater, groundwater, estuarine water, and freshwater samples (Table 1). We treated water samples using a saturated HgCl_2_ solution, 0.45 µm filtration, or the combination of HgCl_2_ and filtration. We stored the treated samples and untreated controls for 0, 1, or 6 months in polypropylene or borosilicate glass vials. To assess the effectiveness of the preservation methods and storage vessels, we compared A_T_ values in all treatments to the respective A_T_ of untreated water from the four sources measured at the beginning of the experiment. To understand whether the amount of HgCl_2_ affects A_T_, we compared concentrations of 0, 0.002, 0.02, 0.05, 0.2, and 0.5% HgCl_2_ on the A_T_ of seawater, groundwater, estuarine water, and freshwater samples stored for 0 and 1 months. Finally, to evaluate whether high dissolved organic carbon (DOC) concentrations that promote biological activity influence the efficacy of different preservation methods, we added glucose to treated (HgCl_2_, filtration) and control water samples, and measured A_T_, DOC, pH, and dissolved oxygen (DO) after 0 and 1 months.

**Table 1:** Location and water parameters for benchmark controls collected from four water sources near Coffs Harbour, New South Wales, Australia. Values in parentheses are standard deviations, n = 5. –, not available.

Type	Location	Date	Total alkalinity (µmol kg^−1^)	Salinity	Temperature (°C)	pH_NIST_	Dissolved oxygen (mg L^−1^)
Seawater	30°16′3.25″S,	8 July 2016	2293.2 (0.6)	33.7	20.8	7.97	9.10
	153°8′15.59″E	24 October 2018	2311.0 (1.5)	34.4	22.4	7.97	8.48
		27 May 2020	2291.2 (1.6)	32.0	21.4	8.14	8.54
Estuarine water	30°17′ 40.55″ S,	12 July 2016	1594.0 (0.8)	18.6	16.4	7.35	8.66
	153° 7′ 3.17″ E	19 December 2018	1296.2 (3.4)	14.2	–	7.65	6.46
		2 June 2020	1798.2 (3.3)	22.0	15.3	7.63	5.93
Freshwater	30° 15′ 5.95″ S,	13 July 2016	327.7 (0.4)	< 0.1	15.3	7.28	9.43
	153° 7′ 53.94″ E	24 October 2018	565.1 (2.7)	< 0.1	20.4	7.09	6.85
		28 May 2020	605.9 (1.6)	< 0.1	17.7	6.53	6.42
Groundwater	30° 17′ 56.69″ S,	14 July 2016	3749.9 (1.5)	20.3	15.1	6.94	5.01
	153° 8′ 3.81″ E	19 December 2018	2722.9 (1.2)	23.1	25.6	6.79	3.96
		3 June 2020	12,382.3 (22.1)	12.7	17.4	7.05	2.61

## Materials and methods

### Study sites and sample collection

Water samples from four sources were collected from locations near Coffs Harbour, NSW, Australia; hereafter called seawater, estuarine water, freshwater, and groundwater. Commonly reported parameters for each water source are shown in Table 1. Water was collected in a 20-L polyethylene drum triple rinsed with the sample water at each location, and transported to the laboratory for processing within 1 h.

### Effects of preservation and storage methods on A_T_

An experiment tested the effects of storage vessel material, preservation method, and storage period on A_T_ using a fully crossed design (2 materials × 4 preservation methods × 3 storage periods), resulting in 24 treatments for each of the four water sources (collected July 2016, Table 1). There were five replicate samples for each treatment combination (24 treatments × 5 replicates per treatment = total 120 independent samples for each water source). The preservation methods were (1) the addition of 100 µL saturated HgCl_2_ solution (25 °C), equivalent to 0.2% of the volume of water samples, (2) filtration using a disposable filter (0.45 µm, Sartorius Minisart NML), or (3) filtration followed by the addition of 100 µL saturated HgCl_2_ solution (25 °C). The control treatment was not filtered and did not have HgCl_2_ added. Treated and control samples were stored in either gas tight glass vials (~ 44 mL, Thermo Fisher Scientific B7950, Type 1, Class A, 33 expansion borosilicate glass) or polypropylene vials (~ 38 mL, Techno Plas P8027UU) for 0, 1, or 6 months.

Vials were prepared by cleaning in a 1 M HCl bath for ~ 24 h, followed by rinsing for ~ 24 h in Milli-Q water (18.2 MΩ cm^−1^ resistivity). Glass vials were then wrapped in aluminium foil and placed in a 450 °C muffle furnace for 4 h to remove organic carbon. Polypropylene vials were dried at room temperature. All vials were tripled rinsed with either the filtered or unfiltered water type according to the assigned treatment, before filling. The vials were filled until a convex meniscus formed and then capped. Capped vials containing samples assigned time 0 were analysed within 3 h of capping. The remaining capped vials were stored in a refrigerator (4 °C) for either 1 or 6 months before analysis to look for changes related to the different processing approaches. Aliquots of the seawater, estuarine water, freshwater, and groundwater (10 mL, n = 5) taken directly from the 20-L drums within 90 min of collection, were analysed (Table 1) and used as benchmark controls to assess changes in A_T_.

### Effects of HgCl_***2***_ concentration on A_T_

An experiment tested the effects of the final concentration of saturated HgCl_2_ in water samples on A_T_ using a fully crossed design (6 HgCl_2_ concentrations × 2 storage periods), resulting in 12 treatments for each of the four water sources (collected October or December 2018, Table 1). There were five replicate samples for each treatment combination (12 treatments × 5 replicates per treatment = total 60 independent samples per water source). All water samples were filtered (0.45 µm, Sartorius Minisart NML) and placed in polypropylene vials (~ 38 mL, Techno Plas P8027UU) as previously described. Aliquots (1, 10, 25, 100, or 200 µL) of saturated HgCl_2_ solution (25 °C) were added, equivalent to 0.002, 0.02, 0.05, 0.2, or 0.5% of the volume of water samples, respectively. A control (0%) treatment did not have mercury added. Initial (0 month) water samples without mercury were used as benchmark controls (water parameters including A_T_ are shown in Table 1). All samples designated time 0 were analysed within 3 h. The remaining vials were stored in a refrigerator (4 °C) for 1 month before analysis.

### Effect of glucose enrichment on the efficacy of preservation methods

An experiment tested the effects of preservation method, water source, and storage period on A_T_ in the presence of high dissolved organic carbon (DOC) levels achieved by the addition of dissolved glucose. High DOC levels promote microbial activity, particularly respiration, which has the potential to alter the carbonate chemistry of stored water samples^22,32^. A fully crossed design was used (3 preservation methods × 2 storage periods × 2 DOC treatments), resulting in 12 treatments for each of the four water sources (collected May or June 2020, Table 1). The preservation methods included the addition of 100 µL saturated HgCl_2_ solution (25 °C), equivalent to 0.2% of the volume of water sample, or filtration using a disposable filter (0.45 µm, Sartorius Minisart NML). A control treatment was not filtered and did not have HgCl_2_ added. A high DOC treatment was created by adding aliquots of a concentrated glucose solution (10,000 ppm, Sigma-Aldrich G8270) to water samples (seawater 48.2 µL; estuarine water 88.8 µL; freshwater 104.3 µL; groundwater 457.9 µL). This treatment increased DOC by an order of magnitude (~ 10–15 times) compared to levels measured in untreated benchmark controls (Supplementary Information Table S1). These DOC concentrations are at the extreme upper limit typically measured in diverse water samples^33^. An ambient DOC treatment did not have glucose solution added.

There were eight replicates for each treatment combination (12 treatments × 8 replicates per treatment = 96 independent samples per water source). Five replicates were used to monitor A_T_ and DOC. To avoid cross-contamination, the remaining three replicates were used to measure pH and dissolved oxygen (DO) at the designated sampling time using a Hach HQ40d multicontroller fitted with a LDO101 DO probe and a PHC301 pH probe calibrated with Metrohm buffers (6.2307.230). Measurements of pH were recorded on the NIST scale (pH_NIST_). Treated and control samples were stored in polypropylene vials (~ 38 mL, Techno Plas P8027UU) for 0 or 1 month, as previously described. Initial (0 month) water samples that were not filtered and did not have mercury or glucose added were used as benchmark controls for A_T_ (water parameters including A_T_ are shown in Table 1). Benchmark controls for DOC, pH, and DO were defined for treated and control water samples (Supplementary information Table S1). All samples designated time 0 were analysed within 3 h. The remaining vials were stored in a refrigerator (4 °C) for 1 month before analysis.

### Sample analyses

Each replicate vial was destructively sampled at its assigned sampling time; for instance, replicates assigned to a 1 month storage treatment were not measured again at 6 months. To measure total alkalinity (A_T_), a 10 mL aliquot from each vial was analysed by potentiometric titration using a Metrohm 888 Titrando^7^, calibrated using certified reference materials (Batch 116 for 2016/17 analyses; Batch 166 for 2018/19 analyses; Batch 170 for 2020 analyses^34^), and titration protocols tailored to each water source developed during previous research (e.g.^2,5^). The protocols ensured the titrations generated sufficient data points by, for example, tailoring the rate at which acid was added to a sample. NaCl was added to the HCl titrant to match the respective salinity of the four water sources (Table 1) (SOP 3^7^). Samples were warmed in a 25 °C water bath prior to analysis, and analyses were carried out in a temperature-controlled room (25 °C). At the designated sampling time (0, 1, or 6 months), all samples from a single water source were analysed in a haphazard order within 3 h after reaching ambient temperature (25 °C). To monitor precision and check for drift, certified reference materials (Batch 116, 166, or 170 respectively) were analysed prior to the commencement of sample analyses and once every 20th sample (every 1–2 h). Across all analyses of reference material, precision was better than 2.3 µmol kg^−1^ (n = 3–5). A_T_ values were calculated using the Gran approach, and, where applicable, corrected for dilution by the HgCl_2_ solution and/or glucose solution^7^. The Gran approach is endorsed by Dickson et al.’s Guide to Best Practice^7^ and the US Geological Survey TWRI Book^35^, is commonly used internationally (e.g.^36–38^), and is the only method suitable for all of the four water sources examined in this study^35^. The Gran approach and curve fitting generate similar alkalinity values, often within 0.1% or 1 µmol L^−1^ (e.g.^39,40^). Any differences between the two calculations are likely less than our error, and would therefore have no material impact on our results or conclusions. Data were used to calculate ∆A_T_ for each replicate, the difference between the A_T_ of the replicate and the mean A_T_ of the respective benchmark control (see Table 1 for A_T_ values of benchmark controls). Standard deviations of ∆A_T_ for each treatment were calculated according to SOP 23^7^.

To measure dissolved organic carbon (DOC), a 3 mL aliquot from each vial was analysed by the wet oxidation method using a OI analytical Aurora 1030 TOC analyser (OI Analytical, USA), with an accuracy of 4% and precision of 2%. Where applicable, DOC values were corrected for dilution by the HgCl_2_ solution^7^. Data were used to calculate ∆DOC for each replicate, i.e. the difference between the DOC of the replicate and the mean DOC of the respective benchmark control. Standard deviations of ∆DOC for each treatment were calculated according to SOP 23^7^.

### Statistical analysis

Dunnett’s T3 tests were used to determine if A_T_, DOC, pH, and DO values in temporal treatments, added HgCl_2_ volume treatments, or added glucose treatments were significantly different from values measured in their respective benchmark control, using IBM SPSS Statistics (v25.0).

## Results

### Effects of preservation and storage methods on A_T_

**Figure 1:**
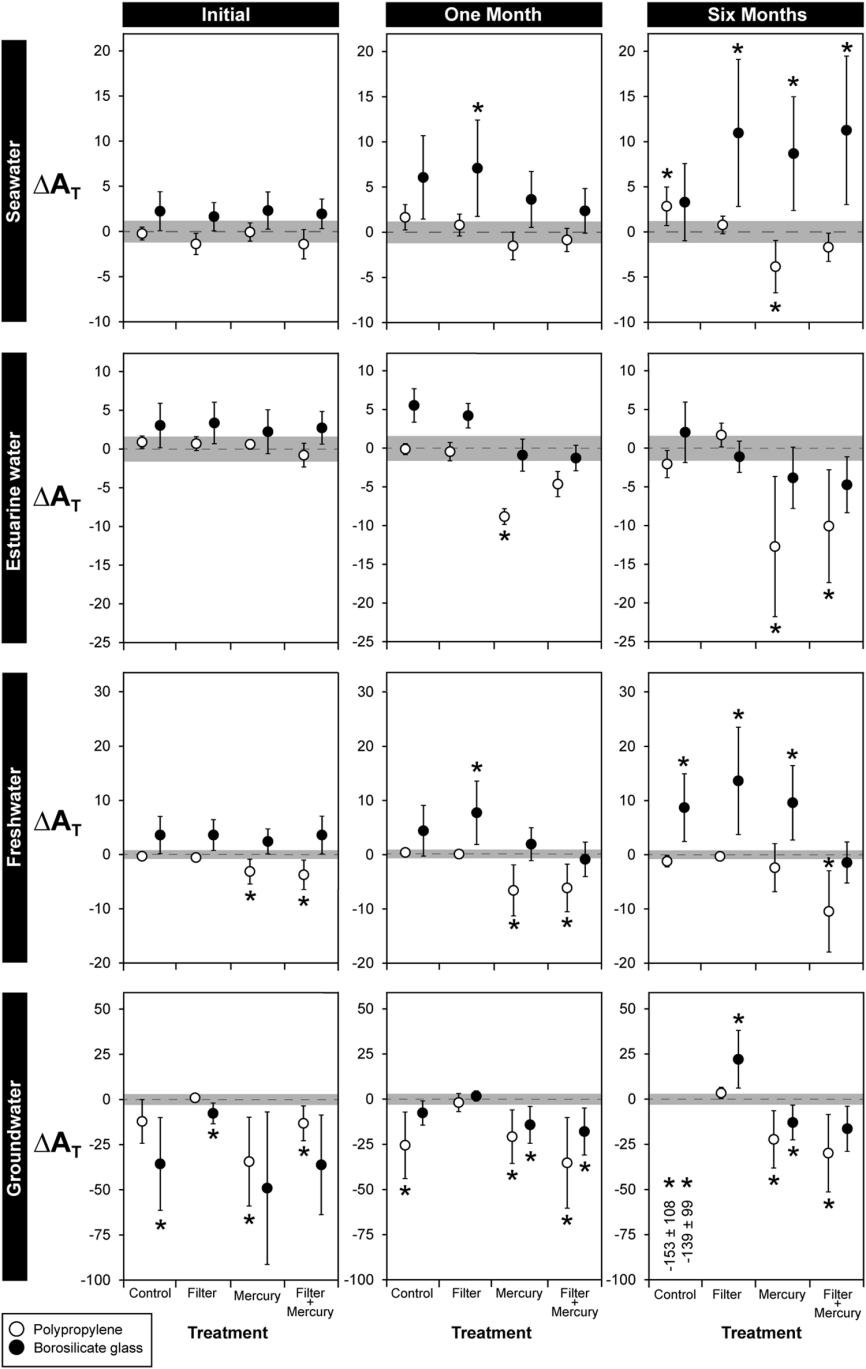
The effects of storage vessel material and preservation method on difference in total alkalinity (∆A_T_) of seawater, estuarine water, freshwater, and groundwater samples stored for 0, 1, and 6 months. All results represent the difference between observations and the mean A_T_ of untreated samples measured at the beginning of the experiment (A_T_ values of benchmark controls shown in Table 1). Water samples were treated using one of four methods (no treatment; 0.45 µm filter; 100 µL saturated HgCl_2_ solution (25 °C); filter + HgCl_2_). Samples were then stored in either polypropylene (white) or borosilicate glass (black) vials at 4 °C for 0, 1, or 6 months. Shaded areas on graphs represent ± 2 standard deviations of the respective benchmark control (Table 1). Asterisks indicate there was a significant difference in A_T_ of samples in a treatment compared to the A_T_ of the benchmark control according to Dunnett’s tests, and should not be used to evaluate statistical difference or similarity among treatments. Data are means ± 1 standard deviation. n = 5 except for the seawater 6 months/glass/Control treatment where n = 4. As mean ∆A_T_ for the groundwater 6 month/Control treatments were greater than –100 µmol kg^−1^, values are given on the figure (means ± 1 S.D.).

The storage vessel and preservation method had significant effects on A_T_ (Fig. 1). Mean A_T_ of freshwater and seawater samples stored in glass vials generally increased over time by 1.6–13.6 µmol kg^−1^ compared to their respective benchmark control (Fig. 1). There were no significant differences in the A_T_ of estuarine water samples stored in glass vials compared to the benchmark control, although mean ΔA_T_ was generally above (after 0 or 1 month) or below (after 6 months) two standard deviations of the benchmark control (i.e. within ± 0.8–3.0 µmol kg^−1^ respectively) (Fig. 1, Table 1). In contrast, the A_T_ of seawater, estuarine water, and freshwater samples stored in polypropylene vials for 0, 1, or 6 months were not different than the A_T_ of their respective benchmark controls, except for mercury and filter + mercury treatments where mean A_T_ was reduced by 0.9–12.7 µmol kg^−1^ compared to the benchmark controls (Fig. 1). For groundwater, the mean A_T_ of samples held in glass and polypropylene vials generally declined by 7.6–153.0 µmol kg^−1^, except for the filter only treatment where A_T_ was generally equivalent to the benchmark control (Fig. 1).

There were no significant differences in A_T_ between control treatments (i.e. no filtration or HgCl_2_) at 0, 1, or 6 months and their respective benchmark control for seawater, estuarine water, and freshwater samples stored in polypropylene (Fig. 1). Conversely, seawater, estuarine water, and freshwater samples held in glass vials experienced increases in A_T_ over time in control treatments by up to 13.6 µmol kg^−1^ after 6 months. For groundwater samples, A_T_ in the control treatments declined regardless of the type of material they were stored in, falling by 3.7–4.1% after 6 months (Fig. 1). For all water sources, mean ΔA_T_ of filtered samples were always comparable to the mean A_T_ of their respective benchmark controls (i.e. within ± 0.8–3.0 µmol kg^−1^ respectively). In contrast, mean A_T_ for all water sources treated with HgCl_2_ or the combination of HgCl_2_ and filtration were generally lower than in benchmark controls by < 49.1 µmol kg^−1^, except for freshwater and seawater samples stored in glass vials where mean A_T_ increased over time by < 11.3 µmol kg^−1^ after 6 months (Fig. 1).

### Effects of HgCl_2_ concentration on A_T_

**Figure 2:**
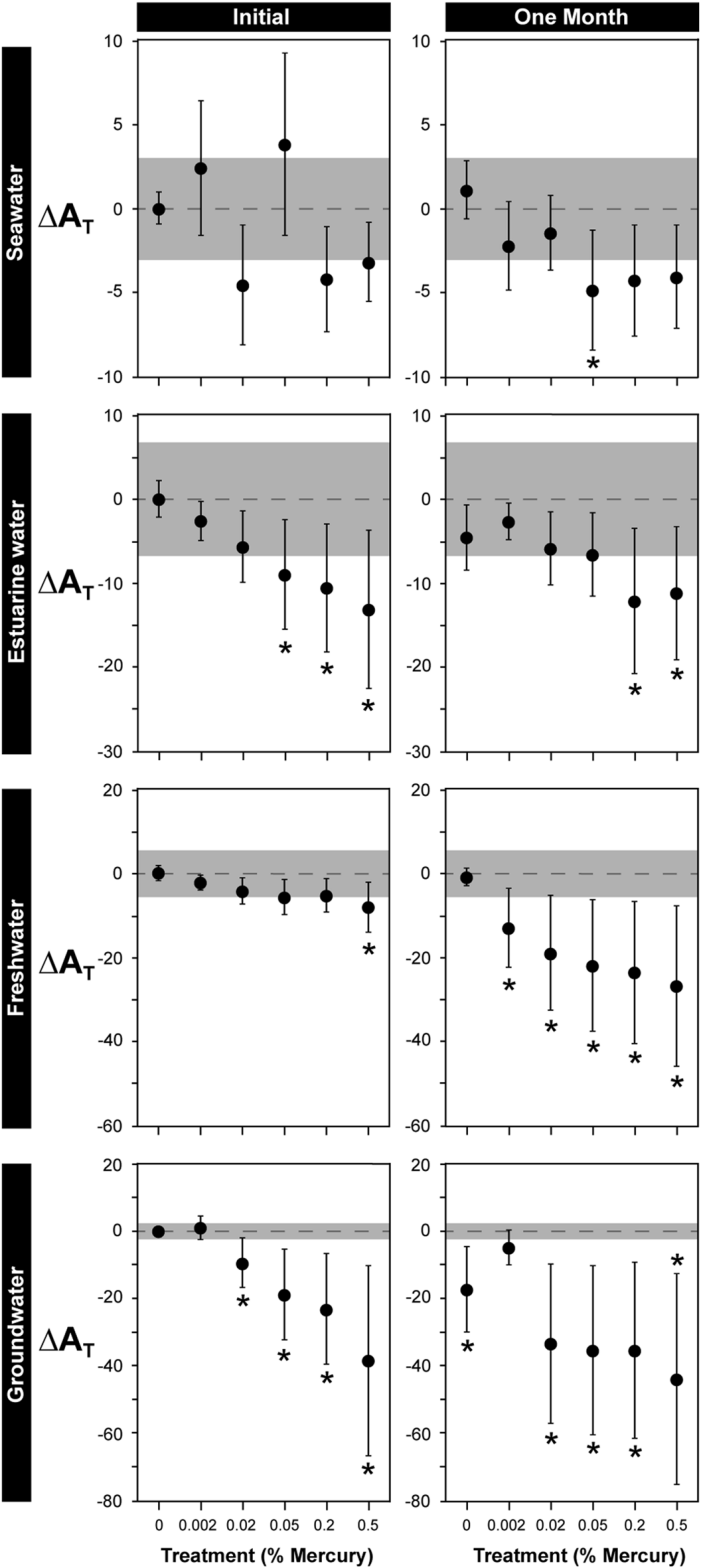
The effects of mercury concentration on total alkalinity (∆A_T_) of seawater, estuarine water, freshwater, and groundwater samples stored for 0 or 1 months. Results represent the difference between observations and the mean A_T_ of untreated samples measured at the beginning of the experiment (A_T_ of benchmark controls are shown in Table 1). Shaded areas on graphs represent ± 2 standard deviations of the respective benchmark control (Table 1). Asterisks indicate there was a significant difference in A_T_ of samples in a treatment compared to the A_T_ of the benchmark control according to Dunnett’s tests, and should not be used to evaluate statistical difference or similarity among treatments. Data are means ± 1 standard deviation. n = 5 except for the freshwater 0.05% mercury/1 month treatment where n = 4.

For estuarine water, freshwater, and groundwater samples, the effects of mercury preservation on A_T_ differed depending on how much saturated HgCl_2_ was added (Fig. 2). Mean A_T_ of estuarine water was reduced by 9.0–13.2 µmol kg^−1^ by the addition of 0.05% or more HgCl_2_ at time 0. After 1 month, estuarine water A_T_ fell by 11.2–12.2 µmol kg^−1^ in 0.2 and 0.5% HgCl_2_ treatments. Mean A_T_ of freshwater was reduced by 8.1 µmol kg^−1^ by the addition of 0.5% HgCl_2_ at time 0, and after 1 month, fell by 13.0–26.8 µmol kg^−1^ in all treatments that had HgCl_2_ added compared to the benchmark control (Table 1). Mean A_T_ of groundwater was always reduced by 9.6–44.1 µmol kg^−1^ by the addition of 0.02% or more HgCl_2_, but there was no difference in the A_T_ of samples with 0.002% HgCl_2_ and benchmark controls. In contrast to the other water sources, mean A_T_ of seawater samples treated with HgCl_2_ were generally not different from the A_T_ of the benchmark control (initial 0% treatment, Table 1), although mean ∆A_T_ of 0.05, 0.2, and 0.5% treatments were generally below two standard deviations of the benchmark control (Fig. 2, Table 1). For all water sources, A_T_ in control treatments without mercury after 1 month were comparable to benchmark controls, except for groundwater where A_T_ fell by 17.5 µmol kg^−1^.

### Effect of glucose enrichment on the efficacy of preservation methods

**Figure 3:**
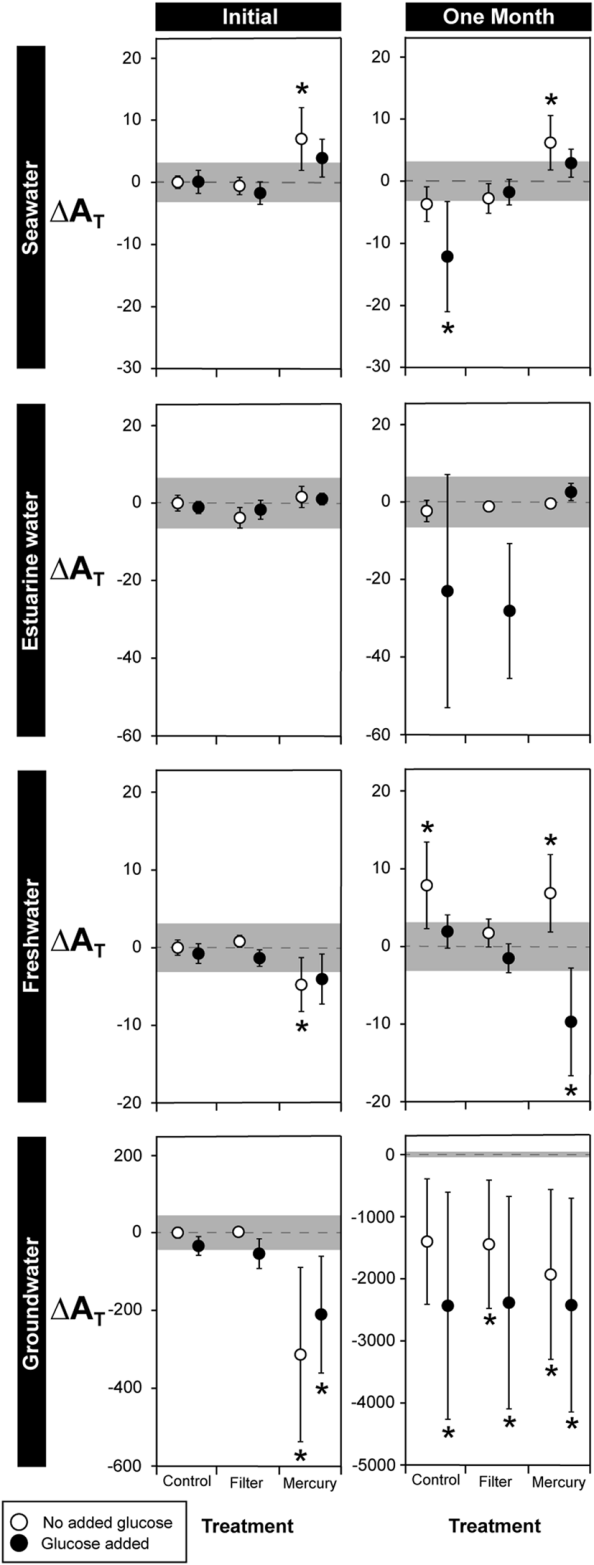
The effects of glucose addition and preservation method on difference in total alkalinity (∆A_T_) of seawater, estuarine water, freshwater, and groundwater samples stored for 0 and 1 month. All results represent the difference between observations and the mean A_T_ of untreated samples measured at the beginning of the experiment (A_T_ values of benchmark controls are shown in Table 1). Water samples were treated using one of three methods (no treatment; 0.45 µm filter; 100 µL saturated HgCl_2_ solution (25 °C)), and had a concentrated glucose solution added (black) or no glucose added (white). Addition of the glucose solution increased dissolved organic carbon (DOC) by ~ 10–15 times compared to ambient levels (Supplementary Information Table S1). Samples were stored in polypropylene vials at 4 °C for 0 or 1 month. Shaded areas on graphs represent ± 2 standard deviations of the respective benchmark control (Table 1). Asterisks indicate there was a significant difference in A_T_ of samples in a treatment compared to the A_T_ of the benchmark control according to Dunnett’s tests, and should not be used to evaluate statistical difference or similarity among treatments. Data are means ± 1 standard deviation. n = 5. Note: scale of Y axes differs for groundwater initial and 1 month.

The addition of glucose to increase dissolved organic carbon (DOC) generally had little effect on all types of samples (Fig. 3, Table 1). For seawater, filtered samples had the same A_T_ as the benchmark control after 0 and 1 month regardless of whether glucose was added (Fig. 3, Table 1). The control treatment also had similar A_T_ to the benchmark control, except after 1 month in the control/glucose added treatment where A_T_ fell by 12.2 µmol kg^−1^. Seawater treated with mercury had higher A_T_ than the benchmark control (∆A_T_ 2.8–7.0 µmol kg^−1^), although this increase was only statistically significant for samples without added glucose (Fig. 3).

For freshwater and groundwater after 0 month, and estuarine water after 0 and 1 month, most treatments had similar A_T_ to the benchmark control (Fig. 3, Table 1). After 1 month, A_T_ in the freshwater control treatment without glucose increased by 7.9 µmol kg^−1^ (Fig. 3, Table 1). Freshwater with HgCl_2_ added had lower A_T_ than the benchmark control at time 0 (∆A_T_ 4.1–4.8 µmol kg^−1^), although this decrease was only statistically significant for samples without added glucose (Fig. 3). After 1 month, freshwater with added HgCl_2_ had either higher (no glucose, |∆A_T_|= 6.8 µmol kg^−1^) or lower (glucose added, |∆A_T_|= 9.7 µmol kg^−1^) compared to the benchmark control (Fig. 3, Table 1).

A_T_ in groundwater samples that had HgCl_2_ added fell by 210.5–313.5 µmol kg^−1^ at time 0 compared to the benchmark control (Fig. 3, Table 1). After 1 month, A_T_ in all groundwater treatments had decreased by 11–20% compared to the benchmark control (Fig. 3, Table 1). Grey-white precipitates were observed in the 1 month control and filtered samples, and black precipitates observed in the 1 month HgCl_2_ treated samples.

There were no clear differences in the effects of preservation treatments on DOC, pH, and dissolved oxygen (DO) regardless of whether samples had glucose solution added (see Supplementary Information). After 1 month, DOC was either the same as or 17–50% lower than the corresponding benchmark control, with decreases in DOC most apparent for groundwater (Supplementary Information Fig. S1, Table S1). However, ∆DOC were generally consistent among preservation treatments for all water sources.

For all water sources, pH and DO in all treatments at time 0 were generally equivalent to levels in the corresponding benchmark controls (Supplementary Information Fig. S1, Table S1). However, estuarine water samples with HgCl_2_ added had significantly lower pH than the corresponding benchmark control (Supplementary Fig. S1, Table S1). After 1 month, pH generally decreased in all treatments by mean 0.1–1.2 pH units regardless of whether samples had glucose added. For all water types, DO generally increased after 1 month by a similar amount in all treatments (0.5–4.3 mg L^−1^), except for groundwater samples with added glucose where DO remained stable across all treatments.

## Discussion

We tested the effects of common storage and preservation methods on the A_T_ of diverse water samples, building on earlier work that focused primarily on seawater samples^7,8^. Estuarine water, freshwater, seawater, and groundwater samples were significantly altered when stored in borosilicate glass vials or treated with HgCl_2_. In contrast, samples filtered to 0.45 µm and/or stored in polypropylene vials for up to 6 months were generally comparable to their benchmark controls. The combination of 0.45 µm filtration and storage in polypropylene vials was the only treatment that consistently prevented changes in A_T_ across most water sources (i.e. within two standard deviations of the benchmark control, ± 0.8–3.0 µmol kg^−1^, respectively), and was equivalent or more effective than HgCl_2_ even when samples were enriched in glucose to promote microbial activity. Based on these results, we contend that filtration and polypropylene are viable alternatives to the use of HgCl_2_ and borosilicate glass for preservation and storage of A_T_ water samples collected from a range of aquatic environments.

The use of poisonous mercury may not be necessary when storing water samples for A_T_ analyses. The addition of saturated HgCl_2_ was often associated with substantial reductions in the A_T_ of freshwater, estuarine water, or groundwater samples stored for 1 or 6 months. It is unlikely that mercury-resistant bacteria reduced A_T_ in these treatments (see^34^) because A_T_ was reduced to the same extent in filter + mercury treatments and mercury only treatments. Instead, Hg^2+^ may have reduced A_T_ by forming complexes with dissolved organic matter (DOM), a component of A_T_^41–43^. DOM interacts strongly with mercury^44^. For example, 45–100% of Hg^2+^ in coastal seawater can be organically complexed with DOM, with the remainder complexed with Cl^−^ or OH^−^ ions^45^. Mercury is more likely to be found in complexes with Cl^−^ than OH^−^ when Cl^−^ levels exceed ~ 350 mg L^−1^, although this is dependent on pH^19,46^. Variability in DOM or Cl^−^ concentrations might therefore explain why the reducing effects of HgCl_2_ on A_T_ in our study were smallest in seawater.

The degree to which HgCl_2_ reduced A_T_ often depended on the concentration used, but this was not consistent among all water sources. The addition of ≥ 0.2% HgCl_2_ to estuarine water and ≥ 0.02% HgCl_2_ to freshwater and groundwater significantly reduced mean A_T_ by anywhere from 0.9% to 4.7% after 1 month. In contrast, A_T_ of seawater was not consistently altered by any of the HgCl_2_ concentrations tested. We are not aware of any studies that have examined the effects of HgCl_2_ concentration on A_T_, but the concentrations that we tested (0.02–0.05%) are often recommended to preserve samples before A_T_ analysis^7^. Our results demonstrate that standard levels of HgCl_2_ used to preserve water samples can reduce the accuracy of A_T_ measurements, particularly for freshwater and groundwater samples, further highlighting the need to identify alternative methods for storing non-oceanic water samples.

Instead of HgCl_2_ preservation, the accuracy of A_T_ analyses can be improved by using filtration to inhibit biological activity in water samples. Filtration has added benefits in that it increases safety for researchers and reduces the costs of managing HgCl_2_ poisoned samples. There was no effect of 0.45 µm filtration on A_T_ of water samples from across a salinity spectrum. Other studies have also found no effects of filtration on the A_T_ of alpine freshwater, pond water, and seawater^6,9,11,21,22^. Importantly there were no changes in the A_T_ of filtered samples stored in polypropylene vials for at least 6 months, with the exception of groundwater in two of three experiments, demonstrating the enduring effectiveness of filtration. Although A_T_ was often unchanged for seawater, estuarine water, and freshwater samples that were not treated with HgCl_2_ or filtered, these water samples should be filtered before storage to prevent changes in A_T_ due to particulates or microbes^20,22^. For some types of groundwater, filtration may not be sufficient to prevent changes in A_T_ over time, although our results indicate changes in A_T_ may be small (< 0.7%) when A_T_ concentrations are < 4000 µmol kg^−1^. We observed precipitates and substantial declines in alkalinity when groundwater samples with very high alkalinity (> 12,000 µmol kg^−1^) were stored for 1 month prior to analysis. We hypothesise chemical or biological activity were responsible for changes in the A_T_ of filtered or unfiltered groundwater, despite refrigeration. Low temperatures slow, but do not stop, chemical and biological activity (e.g.^26^), perhaps also explaining why changes in A_T_ in the groundwater control treatments became more apparent over time (e.g. after 6 months, Fig. 1). For groundwater samples, researchers may need to balance the requirements for accuracy and precision of A_T_ measurements against the risks and costs associated with using combined filtration and HgCl_2_ preservation. If highly accurate measurements are required, our results suggest 0.002% HgCl_2_ can preserve 0.45 µm-filtered groundwater for at least 1 month without substantially altering A_T_, with the exception that waters with extremely high alkalinity should be analysed as soon as practical to avoid physical or chemical changes. Higher concentrations of mercury do not seem to improve preservation.

Polypropylene vials had no measurable effects on the A_T_ of water samples stored for up to 6 months, adding to growing evidence that plastic vessels are suitable alternatives to glassware storage for A_T_ analyses^8,10^. Conversely, some water samples stored in borosilicate glass vials had elevated A_T_, especially in low pH conditions (i.e. pH of groundwater < river < estuary < ocean; Table 1). This is possibly due to the pH-dependent dissolution of acid neutralising materials from the glass (e.g. borate, silicate, or hydroxyl ions^28,47^). The glass vials we used are made to the same specifications as the borosilicate glass bottles recommended by Dickson et al.^7,34,48^, but the glass surface area to water volume ratios are different (our glass vials = 2.0 cm^2^/mL vs. 1-L narrow-mouth bottle = 0.6 cm^2^/mL), which may explain the potential release of detectable amounts of alkalinity in our experiments (also see^28^). Huang et al.^8^ found soda-lime glassware increased A_T_, but reported no effect of borosilicate glass vials on seawater stored for up to 47 days. Differences between our results and Huang et al.^8^ may be because we (i) tested the effects of borosilicate glass using untreated water standards as our benchmarks, (ii) used different brands/shapes of high quality borosilicate glassware that are produced by different manufacturers, which also have different surface area to volume ratios, or (iii) tested for longer storage periods. For example, we found a minor but detectable effect of borosilicate glass on the A_T_ of seawater after 6 months, but not at 0 or 1 month (Fig. 1). The effects of borosilicate glass on A_T_ may also be concealed by the effects of HgCl_2_. When tested in isolation, borosilicate glass and HgCl_2_ had substantial, but opposing, effects on A_T_. In contrast, samples treated with HgCl_2_ and stored in borosilicate glass vials often had equivalent A_T_ to benchmark controls, similar to the generally stable A_T_ of HgCl_2_ poisoned seawater certified reference materials stored in borosilicate glass bottles for up to 3 years^48^. These findings highlight the importance of considering the potential for interactive effects when assessing the efficacy of experimental methods.

The prevention of biological activity that could alter A_T_ is a primary aim of sample preservation methods^7,8^. However, when we added glucose to samples to promote microbial activity, changes in DOC, pH, and DO that could be indicative of biological activity did not substantially differ among treatments over time, nor directly correspond with changes in A_T_ in different preservation treatments. Most changes in A_T_ observed in our experiments were likely due to precipitation, adsorption, flocculation, dissolution, or other chemical reactions. One implication is that preservation and storage methods that are appropriate for stabilising alkalinity may be unsuitable when analysing pH or non-carbonate chemistry parameters where biological activity is a major concern (e.g. DOC, DO). Similar to earlier findings^10,49,50^, filtration and plastic storage vessels were not sufficient to prevent changes in DOC, pH, and DO over time. Consequently, methods to preserve and store water samples need to be tailored to the specific parameter of interest.

Overall, our results suggest there is considerable potential for conventional preservation and storage methods to alter the A_T_ of water samples, particularly from non-marine water sources. To avoid the detectable pitfalls of HgCl_2_ and borosilicate glassware, most water samples intended for A_T_ analysis could instead be filtered to 0.45 µm, and then stored in polypropylene at 4 °C for at least 6 months. Avoiding HgCl_2_ preservation not only improves the precision and accuracy of A_T_ analysis of diverse water types, but also brings environmental benefits, minimises risks to researchers, and ultimately reduces the cost associated with analysis.

## Supplementary Information

Supplementary Information

## Data Availability

All data generated or analysed during this study are presented in this published article and its Supplementary Information file. Datasets are available from the corresponding author on reasonable request.
