# Supplementary material for: Alkalinity of diverse water samples can be altered by mercury preservation and borosilicate vial storage
Source: Sci Rep. 2021 May 11;11:9961. doi: 10.1038/s41598-021-89110-w (PMC8113457; doi:10.1038/s41598-021-89110-w)
Supplement: Supplementary file 1 — Supplementary Information [file 41598_2021_89110_MOESM1_ESM.docx]

**Supplementary Information**

for

**Alkalinity of diverse water samples can be altered by mercury preservation and borosilicate vial storage**

Benjamin Mos^*1^, Ceylena Holloway^1^, Brendan P. Kelaher^1^, Isaac R. Santos^1,2^, Symon A. Dworjanyn^1^

^1^ National Marine Science Centre, Faculty of Science and Engineering, Southern Cross University, Coffs Harbour, New South Wales, Australia.

^2^ Department of Marine Sciences, University of Gothenburg, Gothenburg, Sweden

* **Corresponding author:** Dr. B. Mos

**Email:** benjamin.mos@scu.edu.au

**Number of pages: 4**

**Number of tables: 1**

**Number of figures: 1**

**Table S1.** Initial dissolved organic carbon (DOC), pH measured on the NIST scale (pH_NIST_), and dissolved oxygen (DO) for benchmark controls with and without the addition of a concentrated glucose solution. Water samples were collected from four sources near Coffs Harbour, New South Wales, Australia (Table 1). Values in parentheses are standard deviations. For DOC, n = 5, except for Seawater/Glucose Added and Estuarine water/No Glucose Added where n = 4. For pH_NIST_ and DO, n = 3.

|  | **DOC** |  | **pH_NIST_** |  | **DO** |  |
| --- | --- | --- | --- | --- | --- | --- |
|  | **(µmol L^-1^)** |  |  |  | **(mg L^-1^)** |  |
| **Glucose Added** | **No** | **Yes** | **No** | **Yes** | **No** | **Yes** |
| **Seawater** | 58.7 (1.8) | 1116.4 (32.9) | 8.18 (0.03) | 8.21 (0.01) | 9.02 (0.19) | 9.24 (0.48) |
| **Estuarine water** | 169.6 (6.2) | 2082.8 (121.6) | 7.53 (0.01) | 7.50 (0.01) | 6.11 (0.12) | 5.94 (0.08) |
| **Freshwater** | 231.3 (1.1) | 3013.0 (113.6) | 7.15 (0.04) | 7.00 (0.02) | 7.50 (0.01) | 7.47 (0.04) |
| **Groundwater** | 1003.6 (7.3) | 10095.8 (113.9) | 7.24 (0.01) | 7.28 (0.02) | 0.38 (0.03) | 0.84 (0.30) |


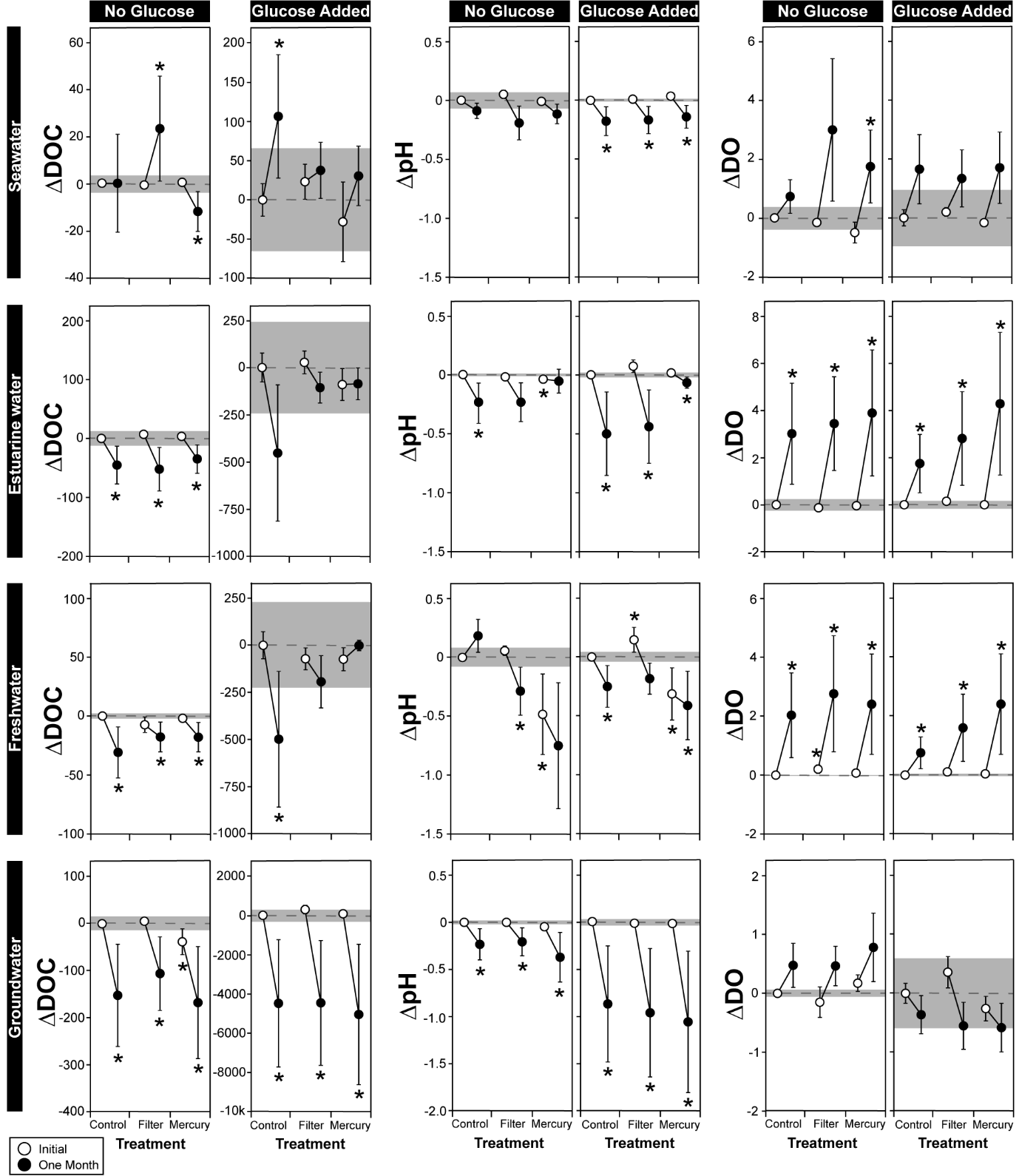


**Figure S1**: The effects of glucose enrichment and preservation method on change in dissolved organic carbon (∆DOC), pH (∆pH), and dissolved oxygen (∆DO) of seawater, estuarine water, freshwater, and groundwater samples stored for 0 (Initial, white) and 1 (black) month. All results represent the difference between observations and the mean value of untreated samples measured at the beginning of the experiment (benchmark controls shown in Supplementary Information Table S1). Water samples were treated using three methods (no treatment; 0.45 µm filter; 100 µL saturated HgCl_2_ solution (25 °C)), and had a concentrated glucose solution added or no glucose added. Samples were stored in polypropylene vials at 4 °C for 0 or 1 month. Shaded areas on graphs represent ±2 standard deviations of the respective benchmark control (Supplementary Information Table S1). Asterisks indicate there was a significant difference in the DOC, pH, or DO of samples in a treatment compared to corresponding values of the benchmark control according to Dunnett’s tests, and should not be used to evaluate statistical difference or similarity among treatments. Data are means ±1 standard deviation. For ∆DOC, n = 4−5. For ∆pH and ∆DO, n = 2−3. Note: scale of Y axes differs among parameters and treatments.
